# A Quality Control Mechanism Coordinates Meiotic Prophase Events to Promote Crossover Assurance

**DOI:** 10.1371/journal.pgen.1004291

**Published:** 2014-04-24

**Authors:** Alison J. Deshong, Alice L. Ye, Piero Lamelza, Needhi Bhalla

**Affiliations:** Department of Molecular, Cell and Developmental Biology, University of California, Santa Cruz, Santa Cruz, California, United States of America; National Cancer Institute, United States of America

## Abstract

Meiotic chromosome segregation relies on homologous chromosomes being linked by at least one crossover, the obligate crossover. Homolog pairing, synapsis and meiosis specific DNA repair mechanisms are required for crossovers but how they are coordinated to promote the obligate crossover is not well understood. PCH-2 is a highly conserved meiotic AAA+-ATPase that has been assigned a variety of functions; whether these functions reflect its conserved role has been difficult to determine. We show that PCH-2 restrains pairing, synapsis and recombination in *C. elegans*. Loss of *pch-2* results in the acceleration of synapsis and homolog-dependent meiotic DNA repair, producing a subtle increase in meiotic defects, and suppresses pairing, synapsis and recombination defects in some mutant backgrounds. Some defects in *pch-2* mutants can be suppressed by incubation at lower temperature and these defects increase in frequency in wildtype worms grown at higher temperature, suggesting that PCH-2 introduces a kinetic barrier to the formation of intermediates that support pairing, synapsis or crossover recombination. We hypothesize that this kinetic barrier contributes to quality control during meiotic prophase. Consistent with this possibility, defects in *pch-2* mutants become more severe when another quality control mechanism, germline apoptosis, is abrogated or meiotic DNA repair is mildly disrupted. PCH-2 is expressed in germline nuclei immediately preceding the onset of stable homolog pairing and synapsis. Once chromosomes are synapsed, PCH-2 localizes to the SC and is removed in late pachytene, prior to SC disassembly, correlating with when homolog-dependent DNA repair mechanisms predominate in the germline. Indeed, loss of *pch-2* results in premature loss of homolog access. Altogether, our data indicate that PCH-2 coordinates pairing, synapsis and recombination to promote crossover assurance. Specifically, we propose that the conserved function of PCH-2 is to destabilize pairing and/or recombination intermediates to slow their progression and ensure their fidelity during meiotic prophase.

## Author Summary

The production of sperm and eggs for sexual reproduction depends on meiosis. During this specialized cell division, homologous chromosomes are linked by at least one crossover recombination event, or chiasma, to promote their proper segregation. How events in meiotic prophase are coordinated to contribute to crossover assurance is not well understood. Here, we show that *C. elegans* PCH-2 regulates a variety of events during meiotic prophase to promote crossover assurance. In the absence of *pch-2*, pairing, synapsis and recombination are accelerated, resulting in defects in synapsis and crossover formation. We propose that PCH-2 restrains the events of meiotic prophase to coordinate them, ensure their fidelity and guarantee that each homolog pair has at least one crossover to promote proper meiotic chromosome segregation.

## Introduction

During sexual reproduction, meiotic chromosome segregation generates haploid gametes so that fertilization restores diploidy to the resulting embryo. Meiosis halves the DNA complement by segregating homologous chromosomes in one division and follows that reductional division with an equational division that resembles mitosis, in which sister chromatids are partitioned. Proper segregation of homologous chromosomes during meiosis I requires the formation of a linkage, or chiasma, between homologs. This linkage is introduced by a series of progressively intimate associations between homologs. Homologs identify and pair with their unique partner. The assembly of a proteinaceous structure, the synaptonemal complex (SC), stabilizes this pairing in a process called synapsis. In the context of synapsis, some programmed double strand breaks (DSBs) are selectively repaired to form crossovers, which give rise to chiasmata. However, how these events are coordinated to guarantee that each homolog pair gets at least one chiasma (the obligate crossover) is poorly understood.

In all meiotic organisms studied, DSBs outnumber crossovers (CO) and can be repaired through multiple mechanisms [1], [2]. Therefore, crossover formation is regulated at multiple points during meiotic DNA repair to assure the obligate crossover. Once a DSB forms, it can either be repaired using the homolog or the sister chromatid as a template. Meiotic DNA repair is biased towards interhomolog recombination to promote the formation of chiasmata [3]. Repair intermediates are then routed through crossover (CO) or non-crossover (NCO) pathways. This decision is thought to be made early, soon after DSB formation [4], and is inhibited by the presence of nearby crossovers (or CO-eligible intermediates), a phenomenon known as crossover interference [5]. Next, a subset of CO-eligible intermediates become crossovers, a process called designation [6]. In addition to these levels of control, feedback or surveillance mechanisms appear to respond to the absence of CO-eligible intermediates [7]–[9]. In *C. elegans*, continued phosphorylation of the nuclear envelope protein SUN-1 is associated with defects in synapsis and recombination, suggesting that this post-translational modification is an indicator of the activation of such a surveillance or feedback mechanism [8], [10].

The role of the conserved meiotic AAA+-ATPase Pch2/TRIP13 has been controversial. In both budding yeast and mice, the *PCH2/Trip13* gene has been implicated at various points in this recombination pathway. Budding yeast *pch2* mutants exhibit elevated rates of DNA repair from sister chromatids [11], [12], misregulation of CO interference in some genetic intervals and an inability to buffer a reduction in DSBs [13], [14]. This is in addition to other reported defects in meiotic chromosome structure [13], [15] and DSB formation [16], [17]. In mice, the requirement for *Trip13* in meiotic chromosome metabolism is conserved [18] and cytological analysis reveals defects in DNA repair and CO formation, distribution and interference in mice deficient in *Trip13* function [19], [20]. Work in *Drosophila* has identified PCH2 as a component of a checkpoint that responds to defects in recombination and meiotic chromosome structure [21], [22]. However, in *C. elegans*, PCH-2 was identified as a component of a checkpoint that monitors synapsis independent of defects in recombination [23]. PCH-2 is conserved in organisms that undergo synapsis during meiosis [24], raising the possibility that the gene product is involved in this process.

AAA-ATPases typically couple ATP hydrolysis to the disassembly of macromolecular complexes [25]. However, the identity of a PCH-2 substrate has remained elusive. Recently, biochemical analysis has revealed that Pch2 purified from budding yeast is capable of removing the meiotic chromosomal protein Hop1 from DNA in an ATP-dependent reaction [26]. Hop1 is a member of the HORMA domain containing family of proteins [27] and is required for pairing, synapsis and inter-homolog recombination [28]–[32]. In vivo, Pch2 may remodel Hop1 on meiotic chromosomes to promote these events [26].

We show that PCH-2 is required to restrain pairing, synapsis and recombination during meiotic prophase. Synapsis and recombination occur more rapidly in *pch-2* mutants than in wildtype worms, producing subtle meiotic defects, and loss of *pch-2* suppresses defects in pairing, synapsis and recombination in some mutant backgrounds. PCH-2 is expressed in germline nuclei prior to meiotic entry. Once meiosis initiates and chromosomes are synapsed, PCH-2 localizes to the SC when homolog-dependent repair mechanisms are active in the worm germline and loss of PCH-2 results in premature loss of homolog-dependent repair mechanisms in mid-pachytene. We conclude that PCH-2 inhibits meiotic prophase events by introducing a kinetic barrier to pairing, synapsis and recombination, potentially by destabilizing their intermediates. In support of this model, defects in synapsis and recombination are less frequent when *pch-2* mutants are incubated at lower temperature and more frequent in both wildtype and *pch-2* mutant worms grown at higher temperature. Since mutation of *pch-2* results in more severe meiotic defects when combined with mutations that abrogate germline apoptosis or mildly affect meiotic DNA repair, we hypothesize that PCH-2 restrains meiotic prophase events to coordinate them, promoting quality control and preventing meiotic defects. We discuss how these findings contribute to our understanding of PCH-2 as a checkpoint protein.

## Results

### PCH-2's role in regulating synapsis and recombination is temperature dependent

**Figure 1:**
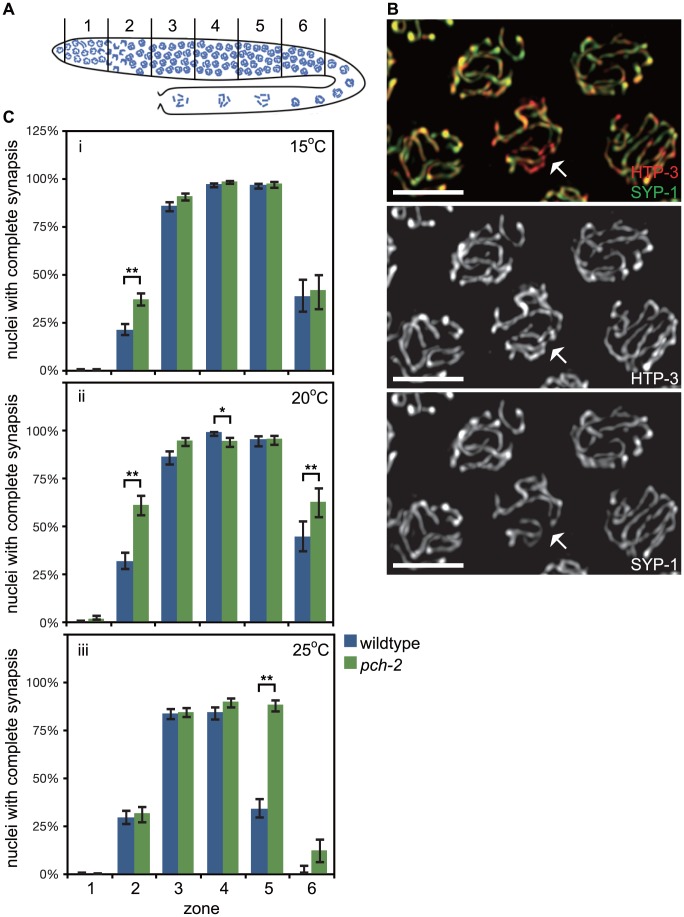
PCH-2's role in regulating synapsis is temperature sensitive. A. Cartoon of the *C. elegans* germline divided into six zones of equal size. In all graphs, meiotic progression is from left to right. B. Meiotic nuclei in wildtype worms stained with antibodies against the axial element component HTP-3 (red) and the central element component SYP-1 (green). Most nuclei exhibit complete colocalization on HTP-3 and SYP-1. One nucleus (indicated by arrow) includes stretches of HTP-3 without SYP-1, indicating the presence of unsynapsed chromosomes. Grayscale images of HTP-3 and SYP-1 are also provided. Unless stated otherwise, scale bars in all images represent 5 microns. C. Histograms representing the percentage of nuclei that exhibit complete synapsis as a function of meiotic progression in wildtype and *pch-2* mutant worms at 15°C (i), 20°C (ii) and 25°C (iii). Error bars indicate 95% confidence interval. For all graphs, a * indicates a p value<0.05 and a ** indicates a p value of <0.0001. Significance was assessed by performing Fisher's exact test.

To determine what role PCH-2 plays in regulating meiotic prophase events in *C. elegans*, we analyzed pairing, synapsis and recombination in the hermaphrodite germline. Meiotic nuclei are arrayed in a spatiotemporal gradient in the germline, allowing for the analysis of the progression of meiotic events as a function of position in the germline (see cartoon in Figure 1A). We divided germlines into six zones of equal size and evaluated the steady-state levels of pairing of the left end of the X chromosome (as visualized by the binding of the protein HIM-8) [33] and the 5S rDNA (as visualized by FISH) in each of these zones and observed no difference in the pairing of these two loci in wildtype and *pch-2* single mutants (Figure S1).

We analyzed SC assembly in wildtype and *pch-2* mutants. The SC is assembled in two steps: axial element proteins, such as HTP-3 [34], [35], load prior to synapsis, and central element components, such as SYP-1 [36], load concomitant with synapsis. Nuclei that have assembled full SC between all chromosome pairs exhibit complete colocalization of HTP-3 and SYP-1 while nuclei with incomplete synapsis have stretches of HTP-3 without SYP-1 (nucleus indicated by arrows in Figure 1B). We calculated the percentage of nuclei with complete synapsis as a function of meiotic progression. Meiotic nuclei in wildtype hermaphrodites grown at 20°C initiated synapsis in the transition zone, corresponding to zone 2, and 99% of meiotic nuclei had completed SC assembly by zone 4 (Figure 1Cii). *pch-2* mutants grown at 20°C also initiated SC assembly in zone 2 but twice as many nuclei completed SC assembly in this early stage of meiotic prophase (Figure 1Cii). Furthermore, nuclei with complete synapsis peaked at 95% in *pch-2* mutants (zones 4 and 5), indicating some slight defects in SC assembly in this mutant background (Figure 1Cii). In zone 6, SC disassembly was also disrupted in *pch-2* mutants grown at 20°C when compared to wildtype worms (Figure 1Cii). Therefore, *pch-2* is required to restrain synapsis early in meiotic prophase and promote SC disassembly in late meiotic prophase when these events occur at 20°C.

In budding yeast, temperature can modify *pch2* mutant phenotypes [13]. We investigated what effect temperature might have on SC assembly and disassembly in *pch-2* mutants by monitoring synapsis at 15°C and 25°C. At 15°C, *pch-2* mutants accelerated synapsis, albeit less dramatically than at 20°C, and exhibited no defects in assembling or disassembling the SC (Figure 1Ci). By contrast, nuclei in *pch-2* mutants grown at 25°C completed SC assembly at frequencies similar to wildtype meiotic nuclei (Figure 1Ciii). The similar progression of SC assembly in wildtype and *pch-2* mutants was accompanied by an increase in nuclei with unsynapsed chromosomes in zones 3 and 4 of the germline in both genotypes (Figure 1Ciii). By zone 4, when the percentage of nuclei with complete synapsis peaked in both wildtype and *pch-2* mutants, 16% and 11% of meiotic nuclei had unsynapsed chromosomes in wildtype and *pch-2* mutant worms, respectively. *pch-2* mutants also disassembled their SCs less efficiently at higher temperature. These data indicate that we can uncouple the acceleration of SC assembly from the defect in SC disassembly by incubating *pch-2* mutants at lower or higher temperature.

**Figure 2:**
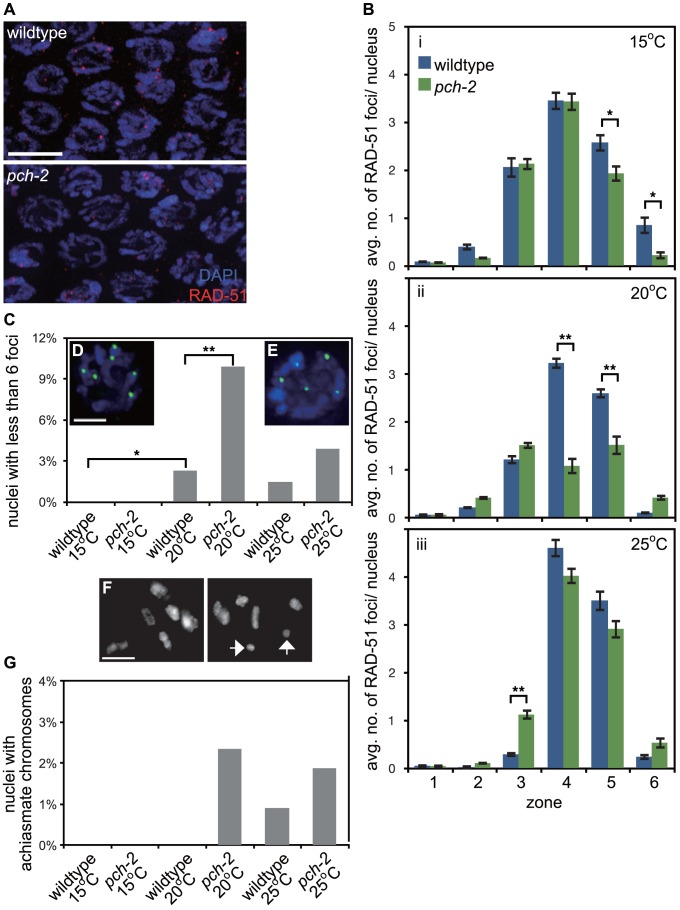
PCH-2's role in regulating recombination is temperature sensitive. A. Meiotic nuclei in early pachytene in wildtype and *pch-2* mutant worms stained with DNA (blue) and antibodies against RAD-51 (red). B. Histograms representing the average number of RAD-51 foci per nucleus as a function of meiotic progression in wildtype and *pch-2* mutant worms at 15°C (i), 20°C (ii) and 25°C (iii). Error bars indicate standard error of the mean. C. Histogram representing the percentage of nuclei with less than six GFP::COSA-1 foci per nucleus in wildtype and *pch-2* mutant worms grown at 15°C, 20°C and 25°C. The number of nuclei assayed for each genotype are as follows: wildtype at 15°C, 303, at 20°C, 304, and 25°C, 339; *pch-2* at 15°C, 318, at 20°C, 293, and 25°C, 360. D. A meiotic nucleus stained with DAPI (blue) with six GFP::COSA-1 foci (green). E. A meiotic nucleus stained with DAPI (blue) with five GFP::COSA-1 foci (green). F. Representative images of late meiotic nuclei without (top) and with (bottom) achiasmate chromosomes (arrows). G. Histogram representing the percentage of meiotic nuclei with achiasmate chromosomes in wildtype and *pch-2* mutant worms grown at 15°C, 20°C and 25°C. The number of nuclei assayed for each genotype are as follows: wildtype at 15°C, 91, at 20°C, 156, and 25°C, 109; *pch-2* at 15°C, 96, at 20°C, 170, and 25°C, 106. For all graphs, a * indicates a p value<0.05 and a ** indicates a p value of <0.0001. Significance was assessed by performing either a paired t-test (2B) or Fisher's exact test (2C).

We dissected how recombination was affected by loss of *pch-2* at different temperatures. First, we assessed the progress of meiotic DNA repair by localizing the recombination protein, RAD-51, in the germlines of wildtype and *pch-2* mutants grown at 15°C, 20°C and 25°C (Figures 2A and B). RAD-51 is required for DNA repair and its presence on chromosomes indicates the introduction of DSBs while its disappearance indicates progression of DSBs through a repair pathway [37]. We determined the average number of RAD-51 foci per nucleus as meiotic nuclei progressed through the germline. At both 15°C and 25°C, average number of RAD-51 foci were similar between wildtype and *pch-2* mutants (Figure 2Bi and iii). However, *pch-2* mutants grown at 20°C had significantly fewer average number of RAD-51 foci in meiotic nuclei zones 4 and 5 than wildtype worms grown at the same temperature (Figure 2Bii).

The reduction in average number of RAD-51 foci on chromosomes in *pch-2* mutants at 20°C could either be the product of a reduction in DSBs or more rapid repair of DSBs. To distinguish between these possibilities, we assayed RAD-51 foci in *rad-54* and *rad-54;pch-2* double mutants (Figure S2). Mutation of *rad-54* prevents the removal of RAD-51 from repair intermediates and stalls the progression of meiotic recombination [38]. *rad-54* single mutants and *rad-54;pch-2* double mutants exhibited a similar profile of RAD-51 loading, both in terms of kinetics and number of foci (Figure S2), indicating that the reduction in average number of RAD-51 foci we quantified in *pch-2* single mutants at 20°C was the product of more rapid repair of DSBs and not a decrease in the introduction of DSBs.

We evaluated how these differences in meiotic DNA repair affected crossover formation. We used GFP::COSA-1 as a cytological reporter to monitor putative crossover formation [6] in wildtype and *pch-2* mutants grown at different temperatures (Figure 2C). Most nuclei in wildtype worms exhibit six GFP::COSA-1 foci (Figure 2D), corresponding to the six pairs of homologous chromosomes that each undergo one crossover [6]. This was particularly striking at 15°C: no nuclei had fewer than six GFP::COSA-1 foci. At 20°C and 25°C, a low percentage of meiotic nuclei in wildtype worms had fewer than six GFP::COSA-1 foci (2.3% and 1.5% respectively) (Figures 2C and E). *pch-2* mutants exhibited a greater range of nuclei with fewer than six GFP::COSA-1 foci. At 15°C, *pch-2* mutants had none, similar to wildtype worms (Figure 2C). However, at 20°C, *pch-2* mutants had a significantly higher fraction of meiotic nuclei with fewer than six GFP::COSA-1 foci than wildtype worms, indicating that crossover assurance mechanisms were compromised in this mutant background. This difference between wildtype and *pch-2* mutants became less severe at 25°C.

To investigate the effects on chiasmata formation, we identified the number of achiasmate chromosomes in wildtype and *pch-2* mutants at each temperature (arrows in Figure 2F). Consistent with our analysis of GFP::COSA-1 foci, there were no achiasmate chromosomes in wildtype and *pch-2* mutants grown at 15°C (Figure 2G). We also did not observe achiasmate chromosomes in wildtype worms grown at 20°C (Figure 2G). *pch-2* mutants grown at 20°C and 25°C had a small percentage of achiasmate chromosomes (2.4% and 1.9% respectively), as did wildtype worms grown at 25°C (1%) (Figure 2G). Thus, our analysis of meiotic DNA repair and crossover formation indicated that performing these tasks at lower temperature suppressed defects observed in *pch-2* mutants grown at 20°C and 25°C. Wildtype worms exhibited defects in these events when incubated at 25°C (Figure 2G).

**Figure 3:**
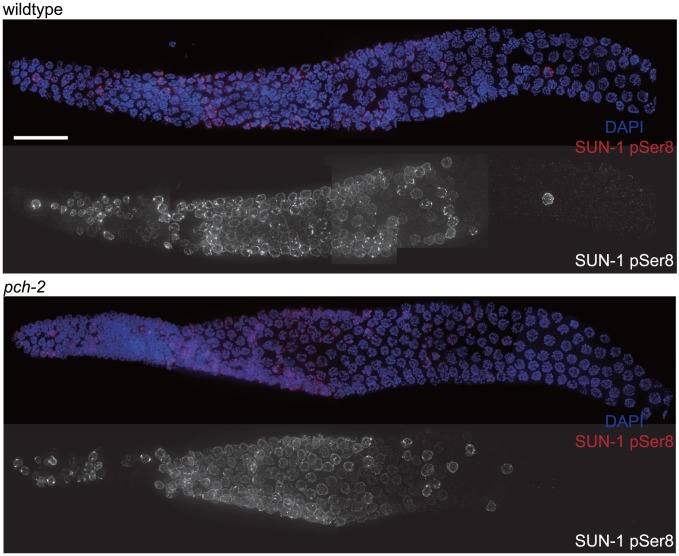
The appearance and disappearance of phosphorylated SUN-1 is not affected by mutation of *pch-2*. Dissected germlines stained with DAPI (blue) and antibodies against SUN-1 pSer8 (red) in wildtype and *pch-2* mutant worms. Grayscale images of SUN-1 pSer8 for both genotypes are also provided. Scale bar represents 20 microns.

Since both synapsis and meiotic DNA repair were accelerated in *pch-2* mutants grown at 20°C, we tested whether meiotic nuclei in *pch-2* mutants entered meiosis earlier or progressed through the germline more rapidly than in wildtype worms at 20°C. Phosphorylation of the nuclear envelope protein SUN-1 occurs at meiotic entry [10], [39] and persists until synapsis is complete and meiotic recombination is proceeding normally [10]. We localized SUN-1 phosphorylated on serine 8 (SUN-1 pSer8) in wildtype and *pch-2* mutants grown at 20°C and did not detect any difference in its appearance or disappearance between the two genotypes (Figure 3).

To determine if nuclei were progressing through the germline more rapidly, we EdU labeled [40] meiotic nuclei in both wildtype and *pch-2* mutants (Figure S3A) and visualized their progression through the various stages of meiotic prophase. Both wildtype and *pch-2* mutants grown at 20°C exhibited a similar progression of EdU labeled nuclei throughout the timecourse (Figures S3B and C). Both of these assays lead us to conclude that the effects on synapsis and recombination that we observed in *pch-2* mutants were not merely the result of more rapid meiotic entry or progression of nuclei through the germline but that PCH-2 is specifically required to restrain these events.

In summary, we report accelerated rates of synapsis and meiotic DNA repair, accompanied by subtle defects in synapsis and recombination, in *pch-2* mutants grown at 20°C (Figures 1Cii and 2Bii). Synapsis and meiotic DNA repair were less affected in *pch-2* mutants cultured at 15°C (Figures 1Ci and 2Bi) and these mutant worms did not have any recombination or synapsis defects (Figures 1Ci, 2C and 2G). Any difference in the rate of SC assembly and meiotic DNA repair between wildtype and *pch-2* mutants was lost at 25°C (Figures 1Ciii and 2Biii) and this correlated with an increase in the frequency of synapsis and recombination defects in wildtype worms (Figures 1Ciii, 2C and 2G). Moreover, defects in SC disassembly in *pch-2* mutants correlated with defects in meiotic recombination (Figures 1Cii, 1Ciii, 2C and 2G).

### Mutation of *pch-2* suppresses pairing defects in the absence of synapsis

Our previous experiments indicated that mutating *pch-2* had subtle effects on synapsis and recombination. To more completely dissect how PCH-2 might be affecting homolog interactions, we investigated the effect of mutating *pch-2* in sensitized mutant backgrounds.

**Figure 4:**
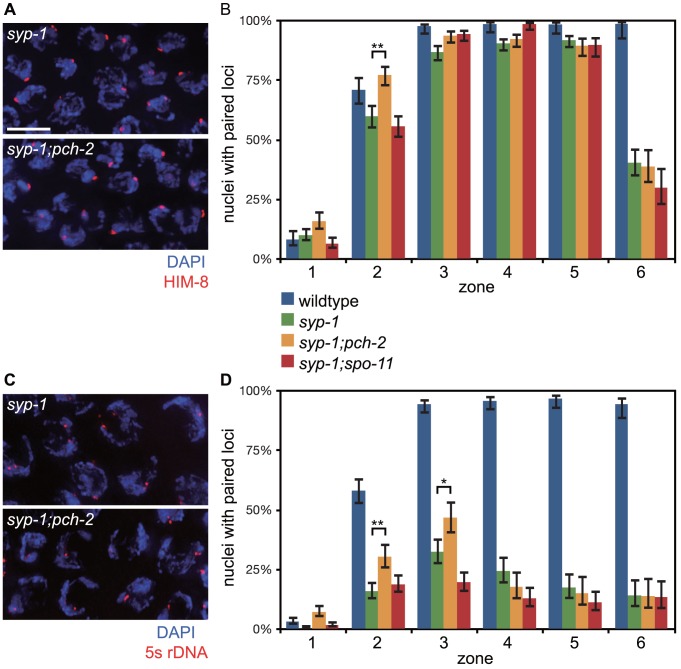
Mutation of *pch-2* suppresses pairing defects in *syp-1* mutants. A. Meiotic nuclei in *syp-1* and *syp-1;pch-2* mutants stained with DAPI (blue) and antibodies against the *X* chromosome PC protein HIM-8 (red). B. Histogram representing the percentage of nuclei with paired HIM-8 signals as a function of germline position in wildtype (from Figure S1A), *syp-1*, *syp-1;pch-2*, and *syp-1;spo-11* mutants. C. Meiotic nuclei in *syp-1* and *syp-1;pch-2* mutants stained with DAPI (blue) and a FISH probe against the 5 s rDNA locus (red). D. Histogram representing the percentage of nuclei with paired FISH signals as a function of germline position in wildtype (from Figure S1B), *syp-1*, *syp-1;pch-2*, and *syp-1;spo-11* mutants. Error bars in both graphs indicate 95% confidence intervals. A * indicates a p value<0.05 and a ** indicates a p value of <0.0001. Significance was assessed by performing Fisher's exact test.

First we analyzed pairing in *syp-1* and *syp-1;pch-2* double mutants. *syp-1* mutants fail to assemble SC between paired homologs, allowing the visualization of pairing intermediates that typically precede and promote synapsis [35], [36]. In *C. elegans*, *cis*-acting sites called Pairing Centers (PCs) are required for efficient pairing and synapsis [35]. In the absence of synapsis, homologous chromosomes exhibit stable pairing of PC ends but not non-PC ends of chromosomes [36]. This interaction depends on both homologs having functional PCs [35]. We monitored synapsis-independent, PC-dependent pairing of *X* chromosomes as a function of meiotic progression by performing immunofluorescence against HIM-8 [41] (Figure 4A). In both *syp-1* single mutants and *pch-2;syp-1* double mutants, PCs of *X* chromosomes initiated pairing in zone 2 and maintained this pairing until late meiotic prophase (zone 6) (Figure 4B). Interestingly, the PC end of *X* chromosomes was more frequently paired in *pch-2;syp-1* double mutants in zone 2 than *syp-1* single mutants, at levels similar to wildtype in the same region of the germline (Figure 4B). Therefore, loss of *pch-2* suppressed the pairing defect of *syp-1* mutants early in prophase. To determine if this effect was an indirect consequence of a reduction in apoptosis affecting the overall population of meiotic nuclei inhabiting hermaphrodite germlines, we also monitored *X* chromosome PC pairing in *spo-11;syp1* double mutants, which display a similar level of germline apoptosis as *pch-2;syp-1* mutants due to abrogation of the DNA damage checkpoint [23]. This double mutant background did not display more stable association of *X* chromosome PCs (Figure 4B), indicating that PCH-2 destabilizes synapsis-independent, PC-dependent pairing.

Loci that are located some distance away from PCs also exhibit synapsis-independent pairing, although to a lesser extent than that observed at PCs [36]. To evaluate the effect of mutating *pch-2* on pairing of an autosomal, internally located locus, we performed FISH against the 5 s rDNA locus (Figure 4C). In *syp-1* single mutants, there was a gradual increase in pairing of this locus until zone 3 (Figure 4D). Once again, in *pch-2;syp-1* double mutants, we observed higher levels of pairing at this locus in both zones 2 and 3 (Figure 4D), indicating that PCH-2 also negatively regulates pairing at a non-PC locus.

### Mutation of *pch-2* suppresses synapsis defects in *meDf2* heterozygotes

**Figure 5:**
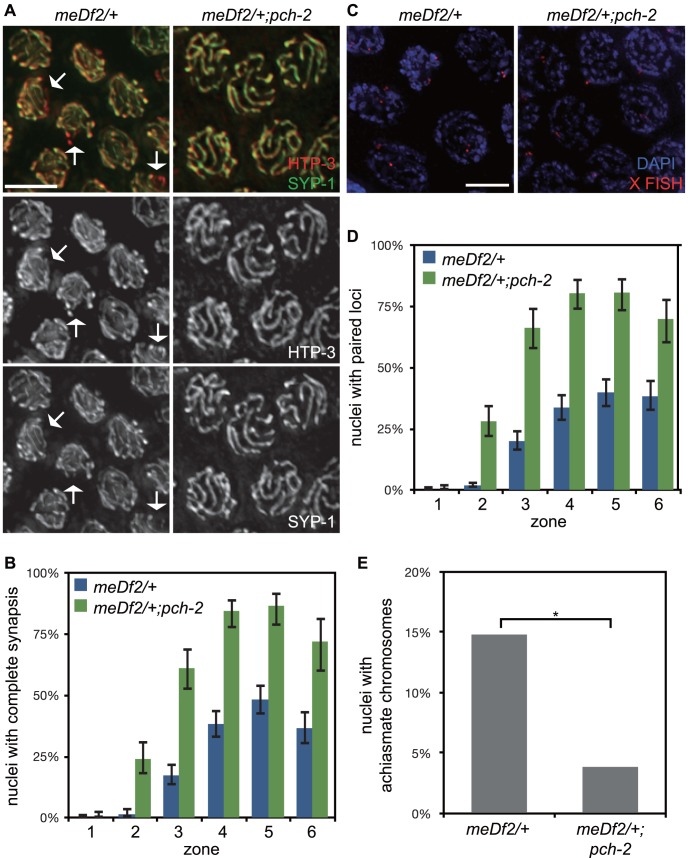
Mutation of *pch-2* suppresses synapsis defects in *meDf2* heterozygotes. A. Meiotic nuclei in *meDf2/+* and *meDf2/+;pch-2* double mutants stained with antibodies against HTP-3 (red) and SYP-1 (green). A fraction of meiotic nuclei in *meDf2/+* have unsynapsed *X* chromosomes (arrows). *meDf2/+;pch-2* double mutants exhibit complete colocalization of HTP-3 and SYP-1, indicating complete synapsis. Grayscale images of HTP-3 and SYP-1 are also provided for both genotypes. B. Histogram representing the percentage of meiotic nuclei that exhibit complete synapsis as a function of meiotic progression in *meDf2/+* and *meDf2/+;pch-2*. C. Meiotic nuclei in *meDf2/+* and *meDf2/+;pch-2* stained with a FISH probe against the *X* chromosome indicates that *X* chromosomes are paired in *meDf2/+;pch-2* mutants. D. Histogram representing the percentage of nuclei with paired *X* FISH signals as a function of meiotic progression in *meDf2/+* and *meDf2/+;pch-2* mutant worms. Error bars in both graphs indicate 95% confidence intervals. E. Histogram representing the number of late meiotic nuclei with achiasmate chromosomes in *meDf2/+* (54 nuclei) and *meDf2/+;pch-2* (78 nuclei). A * indicates a p value<0.05. Significance was assessed by performing Fisher's exact test.

Given that mutation of *pch-2* affected homolog interactions at both PC and non-PC loci, we determined what effect loss of *pch-2* would have on synapsis that could not initiate at two stably paired PCs. *meDf2* is a deficiency that removes the *X* chromosome PC [35], [42]. Animals heterozygous for *meDf2* (*meDf2/+*) exhibit unsynapsed *X* chromosomes in 50% of meiotic nuclei [35] (Figure 5B), illustrating that homolog synapsis can occur, albeit inefficiently, when only one chromosome has a functional PC. We tested whether mutation of *pch-2* in *meDf2* heterozygotes had any effect on the synapsis defect in these hermaphrodites. When we monitored synapsis as a function of meiotic progression in *meDf2/+* animals, there was a gradual increase in meiotic nuclei with complete colocalization of HTP-3 and SYP-1, which mirrored the gradual increase in pairing that has been reported for the *X* chromosome in *meDf2* heterozygotes [35]. By zone 5, 48% of meiotic nuclei had completely assembled SC between all homolog pairs while the remainder exhibit stretches of HTP-3 without SYP-1 (arrows in Figure 5A), which are the unsynapsed *X* chromosomes (Figure 5C). *pch-2;meDf2/+* double mutants also exhibited a gradual increase in the percentage of meiotic nuclei with complete synapsis (Figures 5A and B). However, in this double mutant background, most meiotic nuclei (87%) achieved complete synapsis in zone 5 (Figure 5B), indicating that mutation of *pch-2* suppressed the synapsis defect of *meDf2* heterozygotes. These data explain the reduction in apoptosis we previously reported in *meDf2/+;pch-2* double mutants when compared to *meDf2/+* single mutants [23]. We verified that synapsis was occurring between homologous X chromosomes by performing FISH against a non-PC locus in *pch-2;meDf2/+* double mutants (Figures 5C and D).

We tested whether the synapsis in *pch-2;meDf2/+* double mutants was functional for crossover formation by analyzing the number of nuclei with achiasmate chromosomes in *meDf2/+* single mutants and *pch-2;meDf2/+* double mutants (Figure 5E). Introduction of the *pch-2* mutant allele suppressed the appearance of achiasmate chromosomes in *meDf2* heterozygotes (Figure 5E), indicating that synapsis in *pch-2;meDf2/+* double mutants can support crossover formation.

We next evaluated the percentage of male-self progeny produced by these double mutants. Since males (with a single *X* chromosome) typically arise from spontaneous non-disjunction of *X* chromosomes at a low frequency in wildtype hermaphrodites (with two *X* chromosomes), mutations that increase the frequency of *X* chromosome non-disjunction will exhibit an increase in male self-progeny. For example, *meDf2/+* hermaphrodites produced 11.6% male progeny (Table 1). Consistent with our analysis of synapsis and recombination in *pch-2;meDf2/+* double mutants, these hermaphrodites produced 0.9% male self-progeny (Table 1), indicating that *X* chromosomes segregated correctly during meiosis. Altogether, these data provide additional evidence that PCH-2 normally acts to restrain synapsis, even in situations in which synapsis cannot initiate from two stably paired PCs [35].

**Table 1:** Embryonic viability and frequency of male self-progeny of various mutants.

Genotype	Embryonic Viability	Frequency of male self-progeny
wildtype	100%	0.1%
*pch-2(tm1458)*	100%	0.7%
*mnDp66/+;meDf2/+*	97.5%	11.6%*
*mnDp66/+;meDf2/+;pch-2(tm1458)*	98.2%	0.9%*
*ced-4(n1162)*	90.8%	0.2%
*ced-4(n1162);pch-2(tm1458)*	75.7%	0.2%
*htp-3(vc75)*	100%	0.1%
*htp-3(vc75);pch-2(tm1458)*	81.9%	1.1%

*Counts of male self-progeny were corrected based on the expectation that 12.5% of male progeny and 6.25% of hermaphrodite progeny of these animals are inviable.

### Mutation of *pch-2* exacerbates meiotic defects in mutants defective in germline apoptosis and HTP-3 function

**Figure 6:**
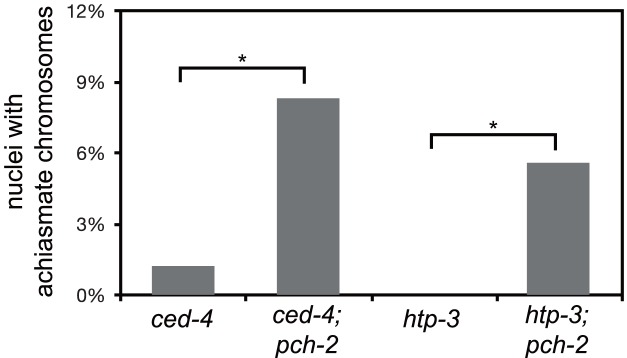
Mutation of *pch-2* exacerbates meiotic defects in mutants defective in germline apoptosis and HTP-3 function. Histogram representing the percentage of late meiotic nuclei in *ced-4* (79 nuclei), *ced-4;pch-2* (68 nuclei), *htp-3* (97 nuclei) and *htp-3;pch-2* (107 nuclei) mutants. A * indicates a p value<0.05. Significance was assessed by performing Fisher's exact test.

Thus far, the acceleration of pairing, synapsis and DNA repair in *pch-2* mutants suggested a role for the gene product in coordinating these events, potentially to promote quality control. If this model is correct, we reasoned that loss of both *pch-2* gene function and other known mechanisms that contribute to quality control during meiotic prophase might increase the frequency of meiotic defects. For example, germline apoptosis removes defective nuclei in late meiotic prophase to prevent the production of aneuploid gametes [23]. To test our hypothesis, we introduced a mutation in *ced-4*, a gene that encodes a core component of the apoptotic machinery that is required for germline apoptosis [43], into *pch-2* mutants and monitored the number of nuclei with achiasmate chromosomes. *ced-4* single mutants had achiasmate chromosomes in 1% of their late prophase nuclei (Figure 6). This frequency of nuclei with achiasmate chromosomes corresponds with the frequency of wildtype meiotic nuclei with less than six GFP::COSA-1 foci (2%), consistent with germline apoptosis culling defective nuclei even during wildtype meiosis. 8% of nuclei in *pch-2;ced-4* double mutants had achiasmate chromosomes (Figure 6), indicating that significantly more meiotic nuclei in *pch-2* mutants have meiotic defects than was represented by the frequency of nuclei with achiasmate chromosomes in the single mutant. Moreover, the increase in meiotic nuclei with achiasmate chromosomes *pch-2;ced-4* double mutants was accompanied by an increase in the production of inviable progeny, indicating the missegregation of chromosomes during meiosis (Table 1).

We wanted to further test our hypothesis that PCH-2 was involved in quality control during meiotic prophase by introducing the *pch-2* mutation into another mutant background to determine if the double mutants would exhibit a more severe meiotic phenotype. However, since *pch-2* mutants suppress some meiotic phenotypes (Figures 4 and 5), we reasoned that we should use a mutation in which the meiotic phenotype was relatively mild, indicating no major defects in meiosis. In this scenario, loss of *pch-2* could reveal a more severe meiotic phenotype.

HORMA domain containing proteins promote many events during meiotic prophase, including pairing, synapsis and recombination between homologous chromosomes [34], [44]–[48]. There are four genes in the *C. elegans* genome that encode meiotic HORMA domain containing proteins (*him-3*, *htp-1*, *htp-2*, and *htp-3*) and recently a hypomorphic allele of *htp-3,vc75*, has been identified. Worms with this mutation appear to undergo meiosis normally, producing no achiasmate chromosomes (Figure 6), but exhibit minor defects in DNA repair and SC disassembly [49]. We generated *pch-2;htp-3(vc75)* double mutants and monitored the viability of its progeny and chiasmata formation in these double mutants. *pch-2;htp-3(vc75)* double mutants had a synthetic meiotic phenotype: Although each individual mutant produced no inviable progeny, the double mutant generated 20% inviable progeny (Table 1). This reduction in viability can be explained by the significant increase in achiasmate chromosomes (Figure 6), indicating that *pch-2;htp-3(vc75)* mutants had a more severe defect in recombination than either single mutant. We ruled out that this synthetic meiotic phenotype could be explained by the possibility that *pch-2* mutants activate an HTP-3 dependent meiotic DNA damage response by analyzing SC disassembly in double mutants. When mutations that activate an HTP-3 dependent meiotic DNA damage response are combined with the *htp-3(vc75)* mutant allele, these double mutants disassemble their SCs more rapidly than the *htp-3(vc75)* single mutant [49]. This did not occur in *pch-2;htp-3(vc75)* double mutants (data not shown). Thus, introduction of the *pch-2* mutation exacerbates the meiotic phenotype of a weak mutant allele of *htp-3*, consistent with a role for PCH-2 in quality control during meiotic prophase.

### PCH-2 localizes to synapsed chromosomes when inter-homolog DNA repair mechanisms are active

**Figure 7:**
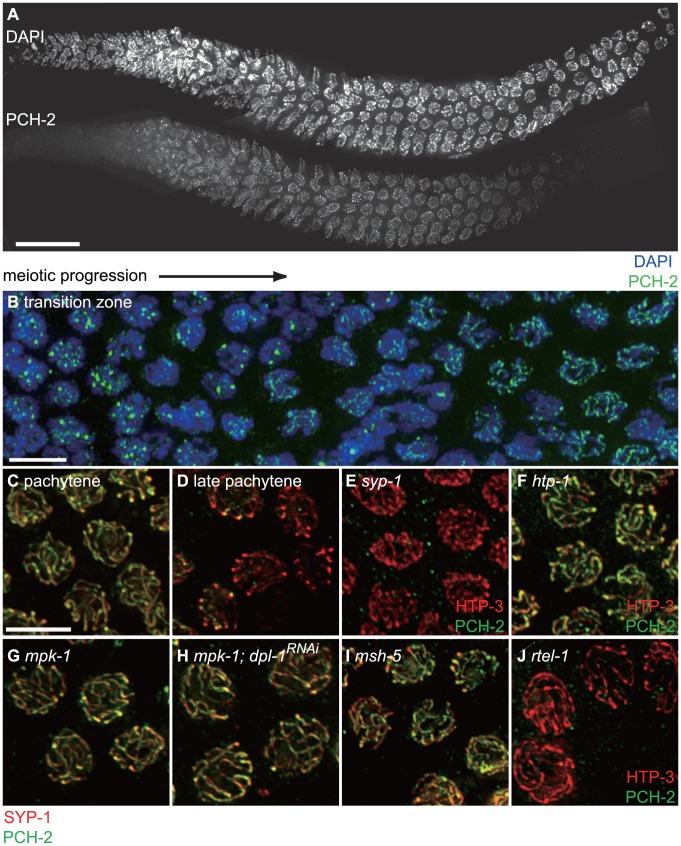
PCH-2 localizes to the SC when inter-homolog DNA repair mechanisms are active. A. A wildtype hermaphrodite germline stained with DAPI and antibodies against PCH-2. PCH-2 is present in germline nuclei prior to the transition zone and removed in late pachytene. Scale bar represents 20 microns. B. Meiotic nuclei in the transition zone stained with DAPI (blue) and antibodies against PCH-2 (green). Meiotic progression for both A and B is from left to right. C. Meiotic nuclei in mid-pachytene stained with antibodies against SYP-1 (red) and PCH-2 (green). PCH-2 colocalizes with SYP-1. D. Meiotic nuclei in late pachytene stained with antibodies against SYP-1 (red) and PCH-2 (green). PCH-2 is absent from meiotic chromosomes. E. Meiotic nuclei in *syp-1* mutants stained with antibodies against HTP-3 (red) and PCH-2 (green). F. Meiotic nuclei in *htp-1* mutants stained with antibodies against HTP-3 (red) and PCH-2 (green). G. Meiotic nuclei in *mpk-1* mutants stained with antibodies against SYP-1 (red) and PCH-2 (green). H. Meiotic nuclei in *mpk-1* mutants in which *dpl-1* has been knocked down by RNAi stained with antibodies against SYP-1 (red) and PCH-2 (green). I. Meiotic nuclei in late pachytene in *msh-5* mutants stained with antibodies against SYP-1 (red) and PCH-2 (green). J. Meiotic nuclei in late pachytene in *rtel-1* mutants stained with antibodies against HTP-3 (red) and PCH-2 (green).

To gain a deeper understanding of how PCH-2 might be regulating pairing, synapsis and recombination, we generated a polyclonal antibody against the PCH-2 protein and localized it in wildtype worms. In wildtype meiotic nuclei, PCH-2 was expressed in germline nuclei before they adopted the polarized morphology that is indicative of meiotic entry, termed the transition zone (Figures 7A and B). In these nuclei, PCH-2 formed foci that did not colocalize with axial elements, such as HTP-3 [34], [35] (data not shown), or with HIM-8 [33] (data not shown). After meiotic entry, PCH-2 localized to meiotic chromosomes in a manner reminiscent of proteins that compose the SC (Figure 7A and B). PCH-2 was loaded into chromosomes in the transition zone (Figures 7A and B) and present in the portion of the worm germline that corresponds to pachytene (Figure 7A). However, unlike SC proteins, PCH-2 was absent from meiotic chromosomes in the most distal region of the germline, corresponding to late pachytene (Figure 6A). To confirm this localization pattern, we co-stained wildtype meiotic nuclei with antibodies against PCH-2 and the SC component SYP-1 [36]. In mid-pachytene, SYP-1 and PCH-2 colocalized (Figure 7C), consistent with PCH-2 localizing to the SC. In late pachytene, SYP-1 was present on meiotic chromosomes but PCH-2 was not (Figure 7D), indicating it was removed from the SC before the SC disassembled.

We tested the genetic requirements for PCH-2 loading on meiotic chromosomes. PCH-2 was not present in germline nuclei when *pch-2* was inactivated by RNA interference in wildtype worms (data not shown). Synapsis is required for PCH-2 localization to the SC, since PCH-2 localization was lost in *syp-1* mutants [36] (Figure 7E). However, homologous synapsis was not, as illustrated by PCH-2's localization to the SC in *htp-1* (Figure 7F) and *sun-1* mutants (data not shown), in which chromosomes synapse non-homologously [46], [50], and in *ieDf2* mutants (data not shown), in which chromosomes self-synapse [51]. PCH-2 was present on the SC when meiotic recombination is disrupted, such as in *msh-5* [52], *zhp-3* [53], *spo-11* [54] (Figure S5), or *rtel-1* mutants [55], [56] (data not shown). We also determined that *htp-3(vc75)* mutants and wildtype animals grown at 25°C exhibited normal localization of PCH-2 (data not shown).

The region of the germline where PCH-2 was absent from meiotic chromosomes is associated with a shift in double strand break (DSB) repair from use of the homolog as a repair template, presumably to use of the sister chromatid [57], [58]. This shift in DNA repair mechanisms is contemporaneous with the loading of the crossover-promoting factor, COSA-1 [6]. To verify that PCH-2's absence from meiotic chromosomes coincided with these events, we performed immunofluorescence on wildtype hermaphrodites harboring a GFP tagged COSA-1 transgene [6] with antibodies against PCH-2. PCH-2 was indeed absent from nuclei that had robust GFP::COSA-1 foci (Figure S4).

The shift in DNA repair mechanisms is dependent on the activity of the MAP kinase pathway [57], the crossover-promoting factor, *msh-5*, and the anti-recombinase, *rtel-1* [58]. PCH-2's removal from meiotic chromosomes in late pachytene did not occur in *mpk-1* (Figure 7G) and *msh-5* mutant backgrounds (Figures 7I and S5) but did in *rtel-1* mutants (Figure 7J). This relocalization also failed to occur when we alleviate the meiotic progression defect in *mpk-1* mutants by RNAi of a downstream effector, *dpl-1* [59] (Figure 7H), indicating that an inability to remove PCH-2 from meiotic chromosomes is not merely an indirect consequence of meiotic prophase arrest in *mpk-1* mutants. PCH-2 was also present on the SC in late pachytene nuclei in *spo-11* and *zhp-3* mutants, suggesting that PCH-2's perdurance is a general response to defects in recombination, similar to the persistence of phosphorylated SUN-1 [10] (Figure S5).

### PCH-2 is required to promote homolog-dependent meiotic DNA repair

**Figure 8:**
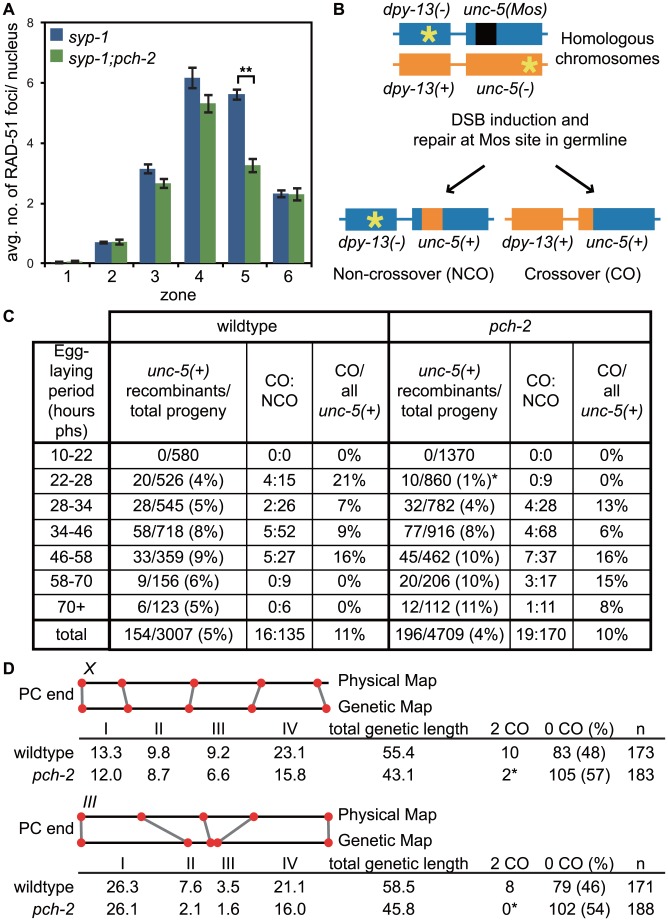
*pch-2* is required to maintain inter-homolog DNA repair in mid-pachytene. A. Histogram representing the average number of RAD-51 foci per nucleus as a function of meiotic progression in *syp-1* and *pch-2;syp-1* mutant worms. Error bars indicate standard error of the mean. A ** indicates a p value<0.0001. Significance was assessed by performing a paired t-test. B. Mos1 excision based DSB repair assay. C. Time-course analysis of inter-homolog recombination induced at the Mos1 site. Chart shows the frequency of *unc-5(+)* recombinants among progeny derived from eggs laid in the indicated time intervals (hours phs) and the frequency of CO and NCO repair types. D. Genetic analysis of meiotic recombination in wildtype and *pch-2* mutants. Physical and genetic maps of Chromosome *III* and the *X* chromosome are depicted to scale. Genetic distance is shown in centimorgans and was calculated as number of recombinants divided by total number of chromosomes assayed. For both C and D, a * indicates a p value<0.05 and significance was assessed by performing Fischer's exact test.

The timing of PCH-2 localization and removal from the SC (Figures 7C and D), along with the effects that mutation of *pch-2* had on meiotic recombination (Figure 2), led us to speculate that PCH-2 might specifically be required to promote homolog dependent DNA repair. To test this, we first monitored the dynamics of RAD-51 loading and removal in *syp-1* and *pch-2;syp-1* mutants (Figure 8A). The complete absence of SC between all homologous chromosomes in *syp-1* mutants [36] results in an inability to repair programmed DSBs because of the absence of closely aligned homologous chromosomes that can be used as repair templates [37]. As a result, RAD-51 foci persist until late pachytene (zone 6 in Figure 8A), when a synapsis-independent repair program is activated. When we compared *syp-1* and *pch-2;syp-1* mutants, we observed that mutation of *pch-2* reduced the average number of RAD-51 foci per nucleus in *syp-1* mutants in zone 5 (Figure 8A). Since our previous experiments ruled out the possibility that mutation of *pch-2* reduced the number of DSBs (Figure S1), these data indicated that some DSBs were being repaired in *pch-2;syp-1* double mutants by synapsis-independent mechanisms, presumably by using the sister chromatid as a repair template. Despite this premature activation of synapsis-independent DSB repair mechanisms, *pch-2;syp-1* double mutants exhibited an extended transition zone [36] and prolonged zone of meiotic nuclei with phosphorylated SUN-1 [10], indicating that meiotic progression is unaffected by mutation of *pch-2* in *syp-1* mutants (data not shown). Moreover, our data also raise the possibility that the partial suppression of recombination defects in *pch-2;syp-1* double mutants is temporally constrained to mid-pachytene (zone 5) since the average number of RAD-51 foci was not significantly different between *syp-1* and *pch-2;syp-1* mutants in earlier zones of the germline (Figure 8A).

We directly tested the role of PCH-2 in promoting homolog-dependent meiotic DNA repair. We used a meiotic recombination assay recently developed to monitor when meiotic chromosomes can access their homolog for DNA repair [58]. This assay takes advantage of an insertion of the *Mos1* transposon into the *unc-5* locus on one homologous chromosome, which disrupts the gene. DSBs are generated in young adult hermaphrodites carrying this locus when heat shock induced expression of the Mos transposase excises the *Mos1* transposon. Since the other homologous chromosome contains a mutation that inactivates the *unc-5* gene elsewhere in the gene, the outcome of DNA repair produces a wildtype *unc-5(+)* allele, allowing these recombinants to be phenotypically identified among the progeny laid by heat-shocked worms. The mode of DNA repair can be determined by monitoring the progeny of *unc-5(+)* recombinants, since the presence of a linked marker (*dpy-13*) can distinguish non-crossover from crossover recombinants (Figure 8B). Given the spatiotemporal organization of meiotic nuclei in the hermaphrodite germline, recombinants laid at different time points reflect the competence of meiotic chromosomes to use their homolog for DNA repair at different stages of meiotic prophase.

When we performed this assay with wildtype and *pch-2* mutant hermaphrodites, we did not recover any recombinants in the first time point (10–22 hours post-heat shock) in both genetic backgrounds (Figure 8C), which corresponds to late pachytene in the hermaphrodite germline when homolog-dependent repair is not active [57], [58]. Given the subtle defects in pairing, synapsis and recombination we observed in *pch-2* mutants (Figures 1, 2 and 3), we divided the next twelve-hour time point (22–34 hours post heat shock) into two six hour timepoints (22–28 hours post heat shock and 28–34 hours post heat shock). Wildtype animals produced recombinant progeny in the 22–28 hour window after heat shock at a frequency of 4%, reflecting the increased competence of nuclei in mid-pachytene for DNA repair from a homologously synapsed partner. In *pch-2* mutants, statistically significant fewer recombinants were generated during this period and of the nine recombinants identified, none were crossovers. In wildtype worms, 21% of recombinants recovered at this timepoint were crossovers.

The difference in the frequency of recombinants between wildtype and *pch-2* mutants was lost in later timepoints (Figure 8C). By the next six-hour interval (28–34 hours post heat-shock), wildtype hermaphrodites generated recombinants at a frequency of 5% and *pch-2* mutants at a frequency of 4%. Wildtype and *pch-2* mutant hermaphrodites generated equivalent frequencies of recombinants until the end of the timecourse. Thus, PCH-2 is required to maintain access to homologous chromosomes for meiotic DNA repair during pachytene.

To determine what effect premature loss of homolog access had on crossover formation in *pch-2* mutants, we monitored recombination genetically in wildtype and *pch-2* mutant animals using five single nucleotide polymorphisms (SNPs) that spanned 95% of Chromosome *III* or the *X* chromosome [60] (Figure 8D). *pch-2* mutants had slightly lower rates of recombination in all intervals across both chromosomes, resulting in a reduction of the genetic length of both Chromosome *III* (from 55.4 to 43.1 cM) and the *X* chromosome (from 58.5 to 45.8 cM). However, the decrease in genetic distance between SNPs was less severe at the PC end of both chromosomes. In addition, mutation of *pch-2* appeared to more strongly affect the genetic distance between SNPs in the center of Chromosome *III*. We observed several double crossovers in wildtype animals on both Chromosome *III* and the *X* chromosome, 8 and 10 respectively. In contrast to wildtype, *pch-2* mutants had significantly fewer double crossovers, only 2 on the *X* chromosome and none on Chromosome *III*. Therefore, the acceleration of homolog-dependent DNA repair in *pch-2* single mutants at 20°C, as assayed both by the appearance and disappearance of RAD-51 foci (Figure 2Bii) and the ability of chromosomes to access their homolog for DNA repair (Figure 8C), reduced the genetic length of chromosomes, albeit not uniformly among genetic intervals, and the frequency of double crossovers.

### Mutation of *pch-2* reduces the percentage of nuclei with six GFP::COSA-1 foci in *meDf2* homozygotes

The localization pattern of PCH-2 in *msh-5* mutants suggested that the persistence of PCH-2 on meiotic chromosomes into late pachytene might be a cytological read-out for prolonged homolog access and that PCH-2 might be required for this extension. However, we could not directly test this possibility in *pch-2;msh-5* double mutants since the assay to monitor homolog access relies on large numbers of viable progeny to assess significance [58]. Instead, we chose to address this possibility in *meDf2* homozygotes. In this mutant, asynapsis of *X* chromosomes produces changes in recombination frequencies in some genetic intervals and a loss of crossover control so that autosomes sometimes experience double crossovers [61]. This redistribution of recombination events in *meDf2* homozygotes could, in part, be the consequence of PCH-2 remaining on meiotic chromosomes in late pachytene and promoting inter-homolog recombination in response to defects in synapsis.

**Figure 9:**
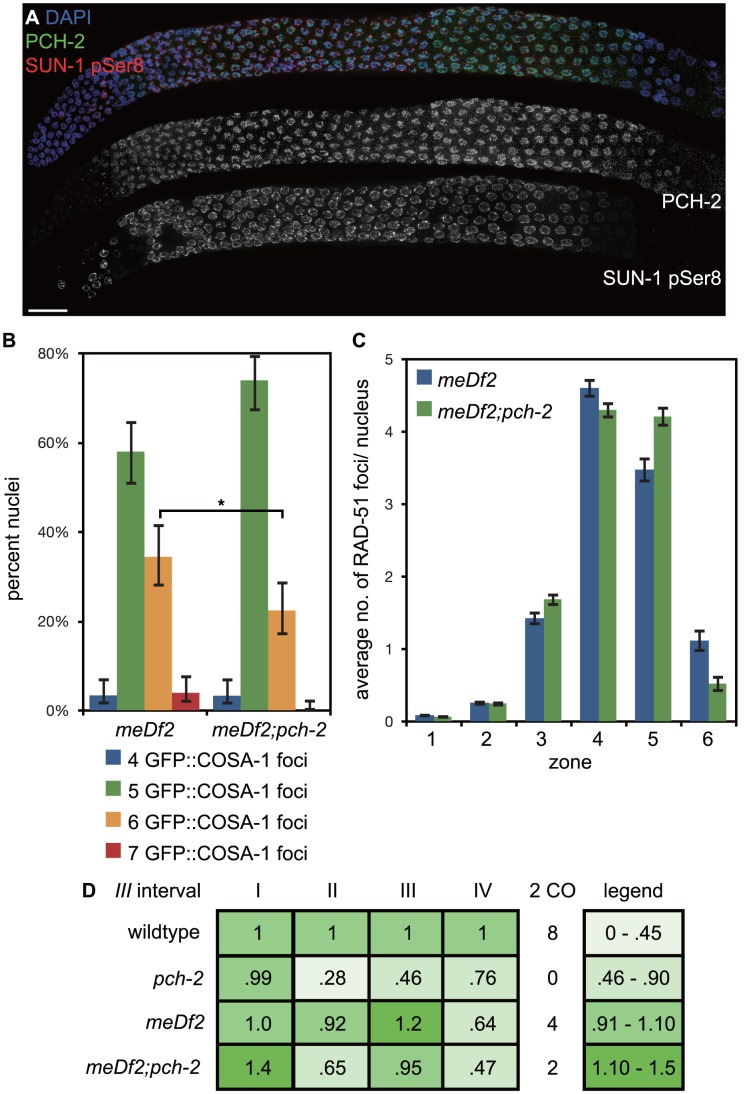
Mutation of *pch-2* reduces the percentage of nuclei with six GFP::COSA-1 foci in *meDf2* homozygotes. A. A dissected germline stained with DAPI (blue) and antibodies against SUN-1 pSer8 (red) and PCH-2 (green). The extension of PCH-2 staining into late pachytene correlates with the extension of phosphorylated SUN-1 staining. Scale bar represents 20 microns. B. Histogram representing the percentage of meiotic nuclei that contain a given number of GFP::COSA-1 foci in *meDf2* and *meDf2*;*pch-2* mutants. 174 *meDf2* nuclei and 178 *meDf2;pch-2* nuclei were counted. Error bars represent 95% confidence interval. C. Histogram representing the average number of RAD-51 foci per nucleus in *meDf2* and *meDf2;pch-2* mutants. Error bars indicate standard error of the mean. For both graphs, a * indicates a p value<0.05. Significance was assessed by performing either Fisher's exact test (7B) or a paired t-test (7C). D. Genetic analysis of meiotic recombination in wildtype, *pch-2*, *meDf2* and *meDf2;pch-2* mutants represented as fractions of wildtype recombination and color-coded according to the legend on the right.

First we determined how PCH-2 localization was affected by asynapsis of *X* chromosomes and whether any change in its localization correlated with characterized responses to asynapsis. We stained *meDf2* hermaphrodite germlines with antibodies against PCH-2 and phosphorylated SUN-1 [10]. The staining pattern for both was extended (Figure 9A), in contrast to their staining patterns in wildtype germlines (Figures 3 and 7A). This extension was accompanied by a delay in the appearance of GFP::COSA-1 foci (data not shown).

Next, we analyzed the number of GFP::COSA-1 foci per nucleus [6] (Figure 9B). In *meDf2* mutants, the majority of germline nuclei (58%) exhibited five GFP::COSA-1 foci. However, a substantial fraction of meiotic nuclei (34%) had six GFP::COSA-1 foci. Even taking into account that 10% of these nuclei had undergone successful synapsis of *X* chromosomes [35], this suggested that about one-quarter of meiotic nuclei had an autosome with an additional putative crossover event. When we analyze GFP::COSA-1 foci in *pch-2;meDf2* double mutants, we observed a statistically significant reduction in the number of germline nuclei that have six foci (22%) and a corresponding increase in the number of nuclei with five foci (74%). Mutation of *pch-2* had no effect on the percent of nuclei with complete synapsis in homozygous *meDf2* mutants (data not shown). These results suggest that cytologically, PCH-2 is required for the increase in putative double crossovers in *meDf2* mutants. However, nuclei with six GFP::COSA-1 foci were still present in *pch-2;meDf2* double mutants, indicating that additional mechanisms promote homolog-dependent meiotic DNA repair in *meDf2* mutants. This possibility was supported by the similar average numbers of RAD-51 foci in both *meDf2* and *meDf2;pch-2* mutants (Figure 9C).

We also assessed double crossover formation genetically in both *meDf2* and *pch-2;meDf2* double mutants on Chromosome *III*. Mutation of *pch-2* did not ameliorate the minor shifts in recombination pattern that we observed in *meDf2*. Indeed, when represented as fractions of wildtype recombination rates, the recombination landscape was more severely affected in *pch-2;meDf2* double mutants than *meDf2* single mutants. There was no difference in the frequency of double crossovers between *meDf2* and *meDf2;pch-2* (Figure 9D), likely because of the removal of meiotic nuclei by germline apoptosis (see Discussion).

## Discussion

Homolog pairing, synapsis and meiosis specific DNA repair mechanisms are essential for the formation of chiasmata and proper meiotic chromosome segregation. Moreover, pairing synapsis and recombination can be temporally and/or mechanistically linked, depending on the organism in question. Therefore, a major challenge during meiotic prophase is to coordinate these events and monitor their progression to ensure that they are occurring appropriately, in the correct order and ultimately give rise to homologous chromosomes linked by at least one crossover.

The acceleration of pairing (Figure 4), synapsis (Figures 1C and 5) and inter-homolog recombination (Figures 2B, 8A and 8C) in *pch-2* mutants results in the loss of crossover assurance (Figures 2C, 2G and Figure 8D), a defect that becomes more severe when germline apoptosis is abrogated or meiotic DNA repair is mildly disrupted (Figure 6). Therefore, we propose that PCH-2 restrains pairing, synapsis and recombination to coordinate them and promote their quality control to assure the obligate crossover. The variability in meiotic defects between *pch-2* mutants and wildtype worms grown at different temperatures suggests that PCH-2 restrains these events by introducing a kinetic barrier to the stabilization of pairing, synapsis and/or recombination intermediates. This barrier is largely restored to *pch-2* mutants undergoing meiosis at reduced temperature (Figures 1Ci, 2C and 2G), suppressing meiotic defects seen in *pch-2* mutants grown at higher temperatures, and overcome in wildtype worms incubated at higher temperature (Figures 1Ciii, 2C and 2G), producing meiotic defects even in the presence of functional PCH-2. A similar observation has been made in budding yeast. *pch2* mutants exhibited fewer defects in recombination when cultured at 30°C than at 33°C [13]. Given PCH-2's membership in a family of proteins characterized by their ability to disassemble macromolecular complexes [25], this kinetic barrier is likely the disassembly of pairing, synapsis and/or recombination intermediates.

The localization of PCH-2 is consistent with this model. PCH-2 is expressed when initial pairing interactions likely occur (Figures 7A and B). Once synapsis has initiated, PCH-2's presence on the SC when meiosis specific homolog-dependent DNA repair mechanisms are active [57], [58] (Figures 7A, C and D) suggests that this localization is related to its regulation of meiotic recombination. Indeed, changes in PCH-2 localization when recombination is defective (Figures 7I, S5 and 9A) also support this interpretation. However, its localization to the SC is not strictly necessary for its effect on recombination, since we still observe a reliance on PCH-2 function in *syp-1* mutants (Figure 8A), which do not assemble SC [36] and fail to localize PCH-2 (Figure 7E). This might suggest that the localization of PCH-2 in nuclei prior to and in the transition zone (Figures 7A and B) could also have implications for the regulation of recombination.

The proposed role of PCH-2 as a factor that disassembles meiotic intermediates is best illustrated by how loss of *pch-2* affects pairing and synapsis. We observe PCH-2's requirement for pairing in *syp-1* mutants (Figure 4), in which the inability to synapse reveals pairing intermediates that contribute to efficient synapsis in *C. elegans* [36]. Homologous PC interactions appear more stable in *pch-2;syp-1* double mutants, indicating that *pch-2* normally destabilizes these interactions and providing a rationale for the accelerated synapsis we observe in *pch-2* single mutants (Figure 1Cii): If efficient synapsis initiates at stably paired homologous PCs [35], enhanced stabilization of PC interactions will promote premature synapsis. However, synapsis can also initiate, albeit less efficiently, in the absence of stably paired PCs, as illustrated by the frequency of synapsis in a mutant in which one homolog has a PC and the other does not (*meDf2* heterozygotes) [35]. Since mutation of *pch-2* suppresses the synapsis defect of *meDf2* heterozygotes (Figure 5) and partially suppresses the pairing defect at a non-PC locus in *syp-1* mutants (Figures 4C and 4D), we support a model in which PCH-2 is required to destabilize pairing intermediates that promote synapsis initiation at both PC and non-PC loci. Indeed, the accelerated synapsis in *pch-2* single mutants could be a consequence of PCH-2 regulating synapsis at both PC and non-PC sites.

We do not favor a model in which meiotic chromosomes in *pch-2* mutants undergo non-homologous synapsis. If accelerated synapsis in *pch-2* mutants produced non-homologous synapsis, we predict that pairing at either PC loci, non-PC loci or both would be significantly reduced. Our pairing analysis in wildtype and *pch-2* mutant worms (Figure S1) does not indicate this. Either loss of *pch-2* does not affect the ability of homologs to identify each other or redundant mechanisms compensate in destabilizing non-homologous interactions.

We hypothesize that loss of *pch-2* also inappropriately stabilizes recombination intermediates, producing an acceleration of meiotic-specific DNA repair, as measured both by the disappearance of RAD-51 from meiotic chromosomes (Figure 2Bii) and the reduced ability to access a homolog as a repair template in mid-pachytene (Figure 8A and 8C). However, we have not directly demonstrated this. It is formally possible that PCH-2*'s* effect on synapsis indirectly affects the progress of meiotic recombination. The ability to uncouple recombination defects (Figures 2C and 2G) from the acceleration of synapsis (Figure 1C) when *pch-2* mutants are incubated at different temperatures and the activation of synapsis-independent DNA repair pathways in *pch-2;syp-1* double mutants (Figures 8A) do not support this interpretation. Moreover, a model in which PCH-2 destabilizes meiotic recombination intermediates could explain the intimate association of PCH2 with early recombination proteins in budding yeast [11] and the phenotypes observed in *pch2* and *Trip13* mutants. Genetically, the inability to disassemble intermediates that are precursors to crossovers may result in additional crossovers and appear as loss of interference in some genetic intervals, as reported in budding yeast *pch2* mutants [13], [14]. Cytologically, this defect would result in an increase in the number of foci containing CO-promoting proteins per chromosome and a loss of cytological interference between these foci, as reported in *Trip13* mutant mice [20]. Consistent with our model, stable recombination intermediates that precede crossover formation, such as the single end invasion intermediate, occur with greater frequency and persist for longer in budding yeast *pch2* mutants [15]. The reason that *pch-2* mutant worms manifest a different recombination defect (fewer crossovers than wildtype) than budding yeast *pch2* mutants or *Trip13* mutant mice (more crossovers than wildtype) is likely due to the dramatically higher levels of DSB formation reported in budding yeast [62] and mice [1], in contrast to *C. elegans* [58].

If PCH-2 destabilizes both pairing intermediates that produce synapsis and recombination intermediates that promote crossovers in *C. elegans*, the question remains whether PCH-2 accomplishes these roles by regulating a single event or factor common to pairing, synapsis and recombination or regulates these events independently of each other. Only the identification of a PCH-2 substrate(s) will directly address this issue. Meiotic HORMA domain containing proteins are very strong candidates, given their role in promoting pairing, synapsis and recombination [34], [44]–[48], their characterized relationship with *PCH2/Trip13* in yeast [13], [15] and mice [10] and Pch2's biochemical activity against Hop1/DNA complexes in vitro [26]. If PCH-2 modulates these events independent of each other, cofactors that contribute to substrate specificity of this particular AAA-ATPase [25] may explain PCH-2's ability to modulate multiple meiotic factors and affect multiple meiotic events.

How does the proposed function of PCH-2 explain its role in meiotic checkpoints? Checkpoint signaling is often initiated from a defective intermediate in the process being monitored. We propose that the synapsis checkpoint is activated by the presence of PCs that are not stably paired and is silenced by stably paired, synapsed PCs. We are not suggesting that the checkpoint is merely monitoring unpaired PCs since homologous PCs appear to pair at significant steady-state levels in *syp-1* mutants but nonetheless activate the synapsis checkpoint [23]. Instead, we propose that the checkpoint specifically responds to the stability of PC pairing. Thus, PCH-2 promotes checkpoint activation by destabilizing paired homologous PCs and inhibiting synapsis. We hypothesize that *pch-2;syp-1* double mutants satisfy the synapsis checkpoint by inappropriately stabilizing paired PCs despite the absence of synapsis, effectively mimicking the stabilization of PC pairing normally accomplished by synapsis. In budding yeast, Pch2 may play a similar role with recombination intermediates that contribute to synapsis initiation, potentially explaining Pch2's initial characterization as a pachytene checkpoint component [63]. Lending support to this hypothesis, budding yeast *pch2* mutants display an increase in synapsis initiation complexes [13], cytologically observed foci that include factors required for both meiotic recombination and synapsis [64], [65].

### PCH-2 promotes homolog dependent DNA repair to ensure the obligate crossover

PCH-2 is required to maintain interhomolog meiotic recombination (Figure 8). A similar role for Pch2 has been demonstrated in budding yeast [11], [12]. However, our experiments reveal that the requirement for PCH-2 in promoting interhomolog meiotic recombination is temporally constrained to mid-pachytene (Figures 8A and C). We cannot interpret any other differences between wildtype and *pch-2* mutants at later timepoints (namely the 58–70 hours phs and 70+ hours phs timepoints) since *Mos* transposase may persist after induction and continue to introduce DSBs throughout the germline.

*Mos*-induced DSBs can be repaired by inter-homolog repair in *pch-2* mutants prior to this window in mid-pachytene, as indicated by the frequency of recombinants laid soon after the 22–28 hour timepoint (Figure 8C). Thus, PCH-2 appears to be redundant with additional mechanisms that promote inter-homolog meiotic recombination earlier in prophase. In addition, *pch-2* mutants do not load GFP::COSA-1 prematurely (data not shown), indicating that switching from meiotic inter-homolog DNA repair to a homolog-independent DNA repair mode can be uncoupled from crossover designation. Alternatively, designation may occur earlier, concomitant with loss of homolog access, and visible COSA-1 recruitment stabilizes the designated crossover.

**Figure 10:**
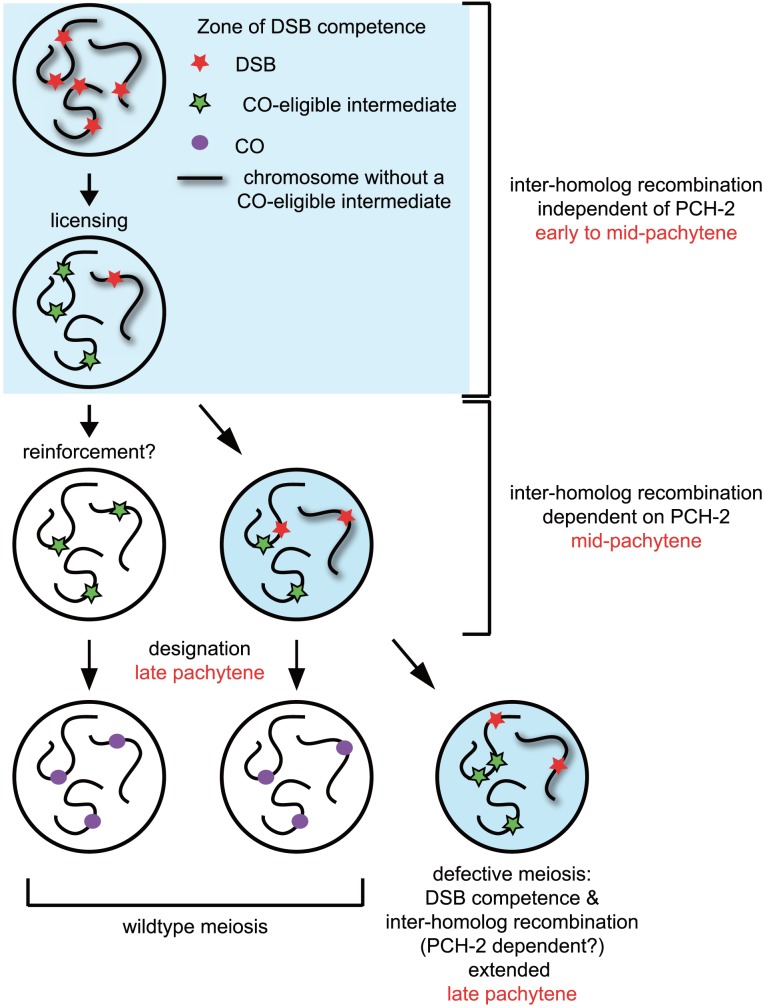
Model for role of PCH-2 during crossover formation. See discussion for details.

Our model of how PCH-2 regulates meiotic recombination is illustrated in Figure 10. Nuclei begin introducing DSBs coincident with the localization of DSB-1 and DSB-2, proteins required for DSB formation and thought to identify a DSB competent period during prophase [8], [9]. A subset of these DSBs become licensed as CO-eligible recombination intermediates [6]. Nuclei in which all chromosomes have a CO-eligible intermediate progress through mid-pachytene and a subset of these CO-eligible intermediates are designated as crossovers in late pachytene [6]. In a small population of nuclei that contain a chromosome without a CO-eligible intermediate, 5–10% based on our studies of GFP::COSA-1 in *pch-2* mutants (Figure 2C), DSB competence is thought to be prolonged to increase the likelihood that a CO-eligible intermediate will be formed. This extension is accompanied by PCH-2-dependent promotion of interhomolog recombination, providing a potential explanation for the presence of double crossovers amongst the progeny of wildtype animals that are lost in the progeny of *pch-2* mutants (Figure 8D).

Why is there a mechanism unique to mid-pachytene to maintain homolog access? Our model assumes the requirement for PCH-2 in mid-pachytene is linked to the maintenance of DSB competence when a chromosome does not have a CO-eligible intermediate. Some possibilities that could explain our results are: 1) The redundant homolog-dependent repair mechanism(s) active earlier in prophase is down-regulated in mid-pachytene, even if DSB competence is maintained. We do not favor this possibility (see below). 2) There is something unique about DSBs or the environment in which DSBs are introduced during mid-pachytene that requires additional reinforcement to promote the formation of crossovers. These models are not mutually exclusive.

We favor another interpretation. Since nuclei in mid-pachytene continue to localize PCH-2 to meiotic chromosomes but do not exhibit other molecular markers associated with DSB competence, such as SUN-1Ser8P [7]–[9] (Figure S6), we suggest that the requirement for PCH-2 in mid-pachytene is independent of DSB competence. This possibility takes into account events that are thought to occur during mid-pachytene. CO-eligible intermediates have been licensed but they have not yet been designated. Villeneuve and colleagues have speculated that a reinforcement step precedes designation, whereby a single CO-eligible intermediate per chromosome is reinforced by CO-promoting mechanisms and ultimately stabilized by designation [6]. Since our analysis of pairing, synapsis and meiotic DNA repair indicates that PCH-2 typically restrains meiotic events, another way to interpret the loss of homolog access in *pch-2* mutants is that it reveals the prematurity of another event, namely reinforcement of crossovers. Since licensed CO-eligible intermediates that are not reinforced are presumably repaired by NCO mechanisms [6], homolog access would be maintained during this process. In the absence of PCH-2 and the more rapid reinforcement of CO-eligible intermediates, homolog access during mid-pachytene would also be lost. This could explain the redistribution of crossovers in *pch-2* mutants (Figure 8D). We think it unlikely that PCH-2 signals the absence of CO-eligible intermediates as its loss does not affect the staining pattern of SUN-1Ser8P [10] and DSB-2 [7]–[9] (data not shown) in genetic backgrounds that have defects in recombination and/or synapsis (i.e. *meDf2* and *syp-1*). Indeed, crossover distribution in *pch-2;meDf2* double mutants appears to be the additive effect of loss of *pch-2* and the delay in meiotic progression introduced by asynapsis (Figure 9D), suggesting that these events are independent of each other.

### Multiple independent feedback mechanisms promote crossover assurance

In contrast to wildtype hermaphrodites, PCH-2 localizes to the SC of meiotic chromosomes in late pachytene when synapsis and/or recombination are defective (Figures 7I, S5 and 9A). This delay in PCH-2 removal correlates with other indicators associated with a delay in meiotic prophase progression, such as phosphorylation of SUN-1 [10] (Figures S5 and 9A) and persistence of DSB-1 and DSB-2 [8], [9] (see nucleus labeled defective meiosis in Figure 10). We suggest that the continued localization of PCH-2 to meiotic chromosomes in mutant backgrounds that perturb synapsis and/or recombination (Figures S5 and 9A) indicates the existence of a feedback mechanism that maintains inter-homolog recombination to promote crossover assurance. Such a model would explain the delay in the appearance of GFP::COSA-1 foci in *meDf2* mutants (data not shown) and the reduction in nuclei with six GFP::COSA-1 foci in *meDf2;pch-2* double mutants (Figure 9B). It would also explain why the “interchromosomal effect” observed in *Drosophila*, the global increase in crossovers produced by chromosomal rearrangements, depends on PCH2 [22]. In contrast to our cytological analysis of GFP::COSA-1 foci, we do not see a corresponding decrease in the number of genetically observed double crossovers in *meDf2;pch-2* double mutants, likely due to small number of double crossovers observed in both mutant backgrounds (Figure 9D). The discrepancy between cytological and genetic evidence of double crossovers could be the result of germline apoptosis.

Since the number and kinetics of RAD-51 focus formation are indistinguishable between *rad-54* and *rad-54;pch-2* (Figure S2), we also conclude that this feedback mechanism is distinct from one that has been proposed to prolong DSB competence in response to defects in synapsis and/or recombination [7]–[9]. PCH-2's proposed role in maintaining interhomolog repair mechanisms in response to defects in meiotic recombination potentially clarifies why *PCH2* is required for crossover homeostasis [13], [14]. A reduction in DSB formation increases the likelihood that chromosomes do not have a CO-eligible recombination intermediate and would activate described feedback mechanisms, including an attempt to increase DSBs [7]–[9] and prolong interhomolog access for repair. Since DSB formation is compromised, there is a greater reliance on maintaining homolog dependent meiotic DNA repair, a pathway (partially) dependent on *PCH2*. This potential relationship between defective meiotic recombination and Pch2's ability to maintain inter-homolog repair into late meiotic prophase could also explain why prophase arrest rescues a reduction in DSB activity but only affects wildtype meiosis minimally [66], especially if Pch2 activity is regulated similarly in budding yeast as in *C. elegans*.

However, mutation of *pch-2* does not eradicate nuclei with greater than five GFP::COSA-1 nuclei in the *meDf2* background (Figure 9B). Moreover, our analysis of RAD-51 in *meD2;pch-2* (Figure 9C) does not indicate the activation of homolog-independent repair mechanisms, in contrast to our studies with *syp-1;pch-2* mutants (Figure 8A). Therefore, we also conclude that at least one other pathway in *C. elegans*, in addition to PCH-2, maintains homolog access in response to defects in synapsis and recombination and that this pathway relies on synapsis for its activity. An attractive candidate for this pathway is the one that we hypothesize acts earlier in meiotic prophase and may be coupled to DSB competence [8], [9].

## Materials and Methods

### Genetics

The wildtype *C. elegans* strain background was Bristol N2 [67]. All experiments were performed at 20° under standard conditions unless otherwise stated. Mutations and rearrangements used were as follows:

*LG I: mnDp66, rtel-1(tm1866), rad-54* *&* *snx-3(ok615), zhp-3(jf61), hT2[bli-4(e937) let-?(q782) qIs48]*

*LG II: pch-2(tm1458), meIs8 [pie-1p::GFP::cosa-1+unc-119(+)]*

*LG III: htp-3(vc75), dpy-5(e61), mpk-1(ga111), ced-4(n1162)*

*LG IV: spo-11(ok79), htp-1(gk174), msh-5(me23), dpy-13(e184) unc-5(ox171::Mos1), unc-5(e791)*, *ieDf2*, *nT1[unc-?(n754) let-?(m435)] (IV, V), nTI [qIs51]*

*LG V: sun-1(jf18), syp-1(me17), krIs14 [hsp-16.48::MosTransposase; unc-122::gfp; lin-15(+)]*

*LG X: meDf2*

Some strains were provided by the CGC, which is funded by NIH Office of Research Infrastructure Programs (P40 OD010440).

### Antibodies, Immunostaining, Fluorescence in situ hybridization, EdU labeling, and microscopy

Polyclonal rabbit antibodies directed against the first 100 amino acids of PCH-2 were generated by Strategic Diagnostic Inc. (SDI, Newark, DE).

Immunostaining was performed as in [23]. Primary antibodies were as follows (dilutions are indicated in parentheses): rabbit anti-SYP-1 (1∶500) [36], guinea pig anti-SYP-1 (1∶500) [36], guinea pig anti-HTP-3 (1∶500) [35], chicken anti-HTP-3 (1∶1000) [35], rabbit anti-RAD-51 (1∶1000) [37], guinea pig anti-SUN-1S8Pi (1∶700) [68] and rabbit anti-PCH-2 (1∶1000). Secondary antibodies were Cy3 anti-chicken, anti-guinea pig and anti-rabbit (Jackson Immunochemicals), Alexa-Fluor 555 anti-rabbit, anti-guinea pig, and anti-chicken (Invitrogen) and Cy5 anti-guinea pig and anti-chicken (Jackson Immunochemicals).

Fluorescence in situ hybridization was performed as described in [33].

Quantification of synapsis, pairing and RAD-51 foci was performed with a minimum of three whole germlines per genotype. For each figure, the number of nuclei assayed for each genotype in each zone is provided in Table S1.

EdU labeling was performed as described in [58]. L4 hermaphrodites were aged for 8–10 hours and moved to plates with *E. coli* labeled with EdU to promote EdU labeling of *C. elegans* meiotic nuclei. A minimum of 15 germlines was scored for each genotype at each timepoint.

All images were acquired using a DeltaVision Personal DV system (Applied Precision) equipped with a 100× N.A. 1.40 oil-immersion objective (Olympus), resulting in an effective XY pixel spacing of 0.064 or 0.040 µm, and a 60× oil-immersion objective (Olympus), resulting in an effective XY pixel spacing of 0.11 or 0.067 µm. Three-dimensional image stacks were collected at 0.2-µm Z-spacing and processed by constrained, iterative deconvolution. Image scaling and analysis were performed using functions in the softWoRx software package. Projections were calculated by a maximum intensity algorithm. Composite images were assembled and some false coloring was performed with Adobe Photoshop.

### Genetic analysis of meiotic recombination

The wild-type Hawaiian CB4856 strain and the Bristol N2 strain were used to assay recombination between single nucleotide polymorphisms (SNPs) on Chromosomes *III* and *X*. The SNPs used on Chromosome *III* were: pkP3081, pkP3095, pkP3101, pkP3035, and pkP3080. The SNPs used on the *X* chromosome were: pkP6139, pkP6120, pkP6157, pkP6161, and pkP6170 [60].

To measure wild-type recombination, N2 males containing *bcIs39* were crossed to Hawaiian CB4856 worms. Cross-progeny hermaphrodites were identified by the presence of *bcIs39* and contained one N2 and one CB4856 chromosome. These were assayed for recombination by crossing with males containing *mIs11* and N2 SNPs. Cross-progeny hermaphrodites from the resulting mate were isolated as L4s, and then cultured individually in 96-well plates in liquid S-media complete supplemented with HB101, carbenicillin, and Nystatin. Four days after initial culturing, starved populations were lysed and used for PCR and restriction digest to detect CB4856 SNP alleles. For recombination in *pch-2* mutants, strains homozygous for the CB4856 background of the relevant SNPs were created, then mated with *pch-2; bcIs39*. Subsequent steps were performed as in the wild-type worms.

### *Mos1* excision-induced DSB repair assay

The *Mos1* excision-induced DSB repair assay was performed as described in [58] with the modification that the second 12 hour window (22–24 hours post-heat shock) was divided into two 6 hour windows (22–28 post-heat shock and 28–34 post-heat shock). Recombinants were identified by their wildtype phenotype and their progeny analyzed to determine whether recombination was the product of a crossover or a non-crossover event. In some cases, whether the recombinants were the products of non-crossover or crossover recombination could not be determined because the recombinant hermaphrodites crawled off the agar and did not produce enough progeny.

## Supporting Information

Figure S1 Pairing is not affected by mutation of *pch-2*. Histograms representing the percentage of nuclei with paired HIM-8 (A) or 5 s rDNA FISH (B) signals as a function of meiotic progression in wildtype and *pch-2* mutant worms. Error bars indicate 95% confidence interval.
(EPS)

Figure S2 DSB formation is similar in *rad-54* and *rad-54;pch-2* mutants. Histogram representing the average number of RAD-51 foci per nucleus in *rad-54* and *rad-54;pch-2* mutants. Error bars indicate standard error of the mean.
(EPS)

Figure S3 Progression of germline nuclei is normal in *pch-2* mutants. A. Dissected germlines in which the fronts of nuclei (blue) with EdU label (red) have reached progressively later stages of meiotic prophase. In all cases, meiotic progression is from left to right. Scale bar represents 10 microns. B. Histograms representing percent of germlines in wildtype and *pch-2* mutants at each time point post-L4 with the EdU-labeled front at the indicated stage. C. Cartoon of hermaphrodite germline with average position of EdU front labeled at various timepoints post-L4.
(EPS)

Figure S4 PCH-2 is absent from meiotic nuclei with GFP::COSA-1 foci. Meiotic nuclei stained with DAPI (blue) and antibodies against GFP::COSA-1 (green) and PCH-2 (red). Grayscale images of DAPI, GFP::COSA-1 and PCH-2 are also provided.
(EPS)

Figure S5 PCH-2 localizes to the SC in late pachytene when meiotic recombination is disrupted. Dissected germlines stained for DNA (blue) and antibodies against SUN-1 pSer8 (red) and PCH-2 (green) in *msh-5*, *spo-11* and *zhp-3* mutant worms. Grayscale images of SUN-1 pSer8 and PCH-2 for all genotypes are also provided. Scale bar represents 20 microns.
(EPS)

Figure S6 The presence of PCH-2 on meiotic chromosomes does not correlate with the presence of phosphorylated SUN-1 in wildtype worms. Wildtype meiotic nuclei stained SUN-1 pSer8 (red) and PCH-2 (green). Meiotic progression is from left to right. Scale bar represents 10 microns.
(EPS)

Table S1 Number of nuclei assayed for each genotype in each zone for all figures. See Materials and Methods for details.
(DOCX)
