# Supplementary material for: A Quality Control Mechanism Coordinates Meiotic Prophase Events to Promote Crossover Assurance
Source: PLoS Genet. 2014 Apr 24;10(4):e1004291. doi: 10.1371/journal.pgen.1004291 (PMC3998905; doi:10.1371/journal.pgen.1004291)
Supplement: Table S1 — Number of nuclei assayed for each genotype in each zone for all figures. See Materials and Methods for details. (DOCX) [file pgen.1004291.s007.docx]

**Table S1: Number of nuclei assayed for each genotype in each zone for all figures.**

|  | | **number of nuclei in each zone** | | | | | |
| --- | --- | --- | --- | --- | --- | --- | --- |
| **Figure** | **Genotype** | **1** | **2** | **3** | **4** | **5** | **6** |
| 1Ci | wildtype | 510 | 501 | 455 | 388 | 314 | 106 |
|  | *pch-2* | 502 | 547 | 493 | 361 | 243 | 129 |
| 1Cii | wildtype | 337 | 334 | 255 | 214 | 172 | 128 |
|  | *pch-2* | 300 | 257 | 262 | 235 | 205 | 126 |
| 1Ciii | wildtype | 379 | 436 | 461 | 307 | 285 | 91 |
|  | *pch-2* | 525 | 477 | 461 | 457 | 367 | 146 |
| 2Bi | wildtype | 134 | 138 | 85 | 100 | 84 | 91 |
|  | *pch-2* | 134 | 135 | 144 | 114 | 114 | 92 |
| 2Bii | wildtype | 167 | 270 | 115 | 178 | 130 | 73 |
|  | *pch-2* | 242 | 219 | 258 | 161 | 105 | 141 |
| 2Biii | wildtype | 139 | 176 | 162 | 120 | 100 | 77 |
|  | *pch-2* | 174 | 163 | 191 | 194 | 133 | 80 |
| 4B | *syp-1* | 359 | 349 | 357 | 433 | 353 | 255 |
|  | *syp-1;pch-2* | 309 | 324 | 303 | 267 | 218 | 104 |
|  | *syp-1;spo-11* | 325 | 395 | 285 | 210 | 177 | 121 |
| 4D | *syp-1* | 342 | 351 | 271 | 213 | 177 | 134 |
|  | *syp-1;pch-2* | 375 | 296 | 203 | 152 | 126 | 108 |
|  | *syp-1;spo-11* | 406 | 378 | 294 | 232 | 195 | 119 |
| 5B | *meDf2/+* | 215 | 202 | 265 | 263 | 244 | 188 |
|  | *meDf2/+;pch-2* | 236 | 245 | 221 | 236 | 198 | 161 |
| 5D | *meDf2/+* | 208 | 233 | 250 | 249 | 231 | 189 |
|  | *meDf2/+;pch-2* | 241 | 218 | 228 | 241 | 204 | 176 |
| 8A | *syp-1* | 195 | 218 | 168 | 182 | 197 | 137 |
|  | *syp-1;pch-2* | 174 | 130 | 136 | 100 | 92 | 66 |
| 9C | *meDf2* | 221 | 219 | 237 | 191 | 156 | 99 |
|  | *meDf2;pch-2* | 283 | 241 | 278 | 207 | 197 | 112 |
| S1A | wildtype | 232 | 219 | 175 | 139 | 127 | 74 |
|  | *pch-2* | 364 | 473 | 388 | 313 | 270 | 174 |
| S1B | wildtype | 407 | 296 | 214 | 188 | 153 | 107 |
|  | *pch-2* | 297 | 326 | 282 | 222 | 179 | 132 |
| S2 | *rad-54* | 148 | 97 | 71 | 56 | 42 | 35 |
|  | *rad-54;pch-2* | 111 | 134 | 120 | 97 | 47 | 45 |
